# Embodiment of emotions in adolescents’ musical expression

**DOI:** 10.1177/03057356241257426

**Published:** 2024-10-08

**Authors:** Suvi Saarikallio, Birgitta Burger, Geoffrey Luck

**Affiliations:** 1 Centre of Excellence in Music, Mind, Body and Brain, Department of Music, Art and Culture Studies, University of Jyväskylä, Jyväskylä, Finland; 2 Institute for Systematic Musicology, University of Hamburg, Hamburg, Germany

**Keywords:** emotional expression, embodiment, music, body movement, adolescence

## Abstract

Music has been actively studied from the perspectives of emotional expression and body movement, but not during adolescence. The current study addressed music as a forum for adolescent embodied emotion expression. Based on prior research, we hypothesised that adolescents would be able to differentiate between emotions in their music-related expressive body movements based on valence and arousal characteristics. Participants (*N* = 60, 17 male, mean age 14.72 years) played djembe to express five basic emotions (happiness, tenderness, sadness, anger, fear) while body movements were motion captured. Correlations of movement features with emotion-regulation tendencies, as measured by the Emotion Regulation Questionnaire (ERQ), were additionally explored. Adolescents demonstrated great capacity to use all measured movement features to express emotions: movement speed, variance, area and length all differed significantly between emotions. In particular, the results confirm the hypothesised connection of high arousal to high speed and acceleration and further suggest that positive valence relates to wider area and longer performance. In addition, adolescents scoring high on cognitive reappraisal gave faster and more stable performances. We discuss creative body movement as part of youth emotional development.

## Musical expression of emotions – conveying affect through sound and body

Musical expression of emotion has been actively studied in recent music psychology research. The vast array of emotional nuances expressed and evoked by music have been addressed through varying theoretical accounts, including basic emotions (e.g., Juslin, 2013; Juslin & Laukka, 2004), dimensional models (Eerola & Vuoskoski, 2013; Rickard, 2004; Salimpoor et al., 2009) and constructionist approaches to emotions (Cespedes-Guevara & Eerola, 2018). Overall, there is a relatively strong consensus about the idea that the core affect dimensions of valence and arousal (the circumplex model of affect, see Russell, 1980) form a feasible grounding for understanding the further shades and layers of musical expression of emotion (Cespedes-Guevara & Eerola, 2018; Eerola & Vuoskoski, 2013). Comprehensive knowledge exists also about how musical features correlate with listeners’ perceptions of the emotional content expressed by the music, especially on the level of the broad emotional categories and dimensions (Eerola et al., 2013; Eerola & Vuoskoski, 2013; Gabrielsson & Lindström, 2010; Juslin & Laukka, 2004). Furthermore, recent theories of embodied cognition stress that emotions inherently involve and are influenced by bodily processes (Carr et al., 2018; Newen et al., 2018; Ward & Stapleton, 2012), and this resonates well with the corporeal nature of musical expression (Leman et al., 2018). Indeed, in most cultures, music and dance are inseparable (Arom, 1991; Nettl, 2000). Yet, while embodiment is inherently present in musical expression of emotion, current knowledge is still relatively limited concerning how particular features of musical movement serve expression of emotional content, especially in adolescence. Here, we investigate how adolescents use musical movement features to express emotions that represent the four quadrants of the circumplex model of affect.

## Body movement and musical movement as emotional expression

Body movement can be considered to be a fundamental aspect of emotional expression. Charles Darwin already noted that joy boosts, but fear hinders movement in animals (Darwin, 1872). Body movement and posture have been shown to express both short-term mood states (Amaya et al., 1996; Castellano et al., 2007; Crane & Gross, 2007; Paterson et al., 2000; Pollick et al., 2001; Troje, 2008; Wallbott, 1998) and long-term emotion-related personality traits (Ball & Breese, 2000; Kluft et al., 1986; Koppensteiner & Grammer, 2010). The same goes for music-induced body movement, which can convey the felt emotions of instrumentalists (Van Zijl & Luck, 2013) and dancers (Saarikallio et al., 2013) as well as be reflective of clinical depression (Punkanen et al., 2017). Body movement can represent emotions that a performer purposefully wishes to convey, and studies have shown that observers are capable of identifying the intended emotions expressed through the movements of dancers and instrumentalists (Camurri et al., 2004; Castellano et al., 2008; Dahl & Friberg, 2007; Thompson & Luck, 2011).

A relatively broad literature has addressed how specific movement patterns in general relate to different emotional contents (Coulson, 2004; Dael et al., 2012; Wallbott, 1998). Dael et al. (2012) asked actors to portray 12 different emotions through their body movement and observed that the action patterns of different emotions were reflective of the broader functional components of emotion such as modes of appraisal and action readiness. Overall, the increases or decreases in the amount of movement can be broadly connected to approach vs. avoidance motivation, a theoretical distinction concerning emotional behaviour. Approach motivation (behavioural activation system, extraversion, positive emotionality) relates to an increase in behavioural activation towards a desired goal, while avoidance motivation (behavioural inhibition system, negative emotionality, neuroticism) relates to a decrease in behavioural activation caused by an undesired event or goal (Elliot & Thrash, 2002; Gray, 1970). Research has shown that emotions characteristic of high arousal and positive valence (indicative of approach motivation) are conveyed by faster and broader movement, while emotions characteristic of low arousal and negative valence (indicative of avoidance motivation) are conveyed via decreased movement (Amaya et al., 1996; Paterson et al., 2000; Pollick et al., 2001; Wallbott, 1998). Differences in reactions to stimuli representing the approach‒avoidance continuum are present already in 6-month-old babies, who demonstrate higher interest/preference to happy than sad music excerpts, as indicated by greater length of turning their gaze towards the happy stimuli (Nawrot, 2003).

Arousal level in particular tends to be reflected in the speed of movement. Faster movements are characteristic of anger, joy and excitement, while slower movements are more representative of sadness, tiredness and other low-arousal emotions (Amaya et al., 1996; Castellano et al., 2007; Paterson et al., 2000; Pollick et al., 2001; Wallbott, 1998). Posture may be particularly important in the perception (or expression) of valence, with open and upright postures relating to positive emotional states and forward-leaning and more closed postures being connected with sadness, shame and boredom (Castellano et al., 2007; Coulson, 2004; De Silva & Bianchi-Berthouze, 2004; Schouwstra & Hoogstraten, 1995; Wallbott, 1998).

Studies of music-induced movement support the observations of the general body movement research. Personality, for instance, is related to particular features of music-induced movement: Individuals scoring high on extraversion demonstrate faster, more varied dance movements with greater use of space and body extremities, while individuals high on neuroticism demonstrate slower, less-varied movements, limited use of space and less movement of the extremities (Luck et al., 2010). Current mood state reflective of approach motivation (positive, energetic state) is related to faster, more varied (higher acceleration) and more complex movement patterns and a more open posture, particularly in terms of hand movement (Saarikallio et al., 2013). Both these studies demonstrate a connection between positive emotion and greater amount of movement. Conversely, clinical depression is related to a reduced speed, variance and amount of music-related dance movement across a range of body parts (Punkanen et al., 2017).

Observations from musical movement share similarities with how emotion perception is grounded in acoustic features of music and sound (Bowling et al., 2012). The clearest corresponding aspect is emotional arousal, which relates to both tempo of music and speed of movement: High-arousal emotions of happiness, anger and fear are typically conveyed by faster tempo, while low-arousal emotions of sadness and tenderness are characterised by slower tempo (Eerola et al., 2013; Gabrielsson & Lindström, 2010; Juslin & Laukka, 2004). Adult laypersons can transfer the emotional content of music into expressive body movement at least on the level of broad emotion categories such as happiness, sadness, anger and tenderness (Burger et al., 2013; Burger & Toiviainen, 2020), and even 4-year-olds can do this to differentiate between happiness and sadness (Boone & Cunningham, 2001).

## Adolescents’ expression of emotion through musical movement and the broader emotional skills

The current paper focuses on adolescents. Many studies have shown that adolescents actively use music for their emotional needs such as mood management and consolation (e.g., North et al., 2000; Saarikallio & Erkkilä, 2007; Ter Bogt et al., 2017). Meanwhile, only a limited number of studies have focused on their emotional expression through sound and movement (Davis et al., 2020), and better understanding of adolescents’ embodied emotion expression in music might enrich knowledge about their broader emotional development and the role of music in it. It has been shown that adolescents have insightful understanding of music being expressive of emotions and that their perception and interpretation of musical emotions also develop across the ages of 10–18 years, shifting from more self-referential meaning-making towards a more detached and evaluative stance (Herbert & Dibben, 2018). Adolescents’ perception of emotions in movement also changes: For instance, the use of velocity cues to perceive emotions from the movement of others is influenced by the adolescent’s own typical walking speed, which slows down across the ages of 11–18 years (Edey et al., 2020). It has also been demonstrated that the bodily sensation patterns of emotions develop towards more adult-like patterns from age 6 to 17 years, in parallel with the use of emotion words (Hietanen et al., 2016). The link between bodily and conceptual levels of self-awareness has also been observed in a study by Georgiou et al. (2016), who showed that the difficulties of 12- to 17-year-olds in describing feelings (a facet of alexithymia as measured by Toronto Alexithymia Scale, TAS-20) were related to higher susceptibility for illusions in body-ownership (the rubber hand illusion; Georgiou et al., 2016).

The capacity to express emotions is one of the key elements of broader social-emotional competence, which further includes competencies such as emotion regulation and relationship skills (Denham et al., 2012; Halberstadt et al., 2001; Mayer & Salovey, 1997; Saarni, 1999). Different components of emotional competence are intercorrelated. For instance, difficulties in differentiating between negative emotions are related to difficulties in regulating negative emotions (Feldman Barrett et al., 2001). The emotion-regulation capacities of adolescents then further mediate the connection between emotional awareness and potential risk for detrimental health outcomes such as depression (Van Beveren et al., 2019). Adaptive emotion-regulation strategies (e.g., reappraisal, distraction) prevent undesirable developmental paths towards social and mental problems (Zeman et al., 2006). In contrast, deficits in emotion regulation – and the use of maladaptive strategies (e.g., rumination, avoidance) – are prominent in the development and maintenance of many forms of psychopathology, including mood and personality disorders (Berking & Wupperman, 2012; Gross, 2002). One example of a broadly used and validated tool for measuring competence in emotion regulation is the Emotion Regulation Questionnaire (ERQ, Gross & John, 2003), which assesses a person’s tendency to use two different regulation strategies: *cognitive reappraisal*, which refers to modulating one’s thinking and re-interpreting emotional events to change their emotional impact (Gross & John, 2003; Lazarus & Alfert, 1964) and is connected to positive health outcomes, and *expressive suppression*, which refers to modulating one’s behavioural responses to emotional events through attempts at hiding, inhibiting or reducing the ongoing emotion-expressive behaviour (Gross & John, 2003; Gross & Levenson, 1993) and is less advantageous to wellbeing (for a review, see the article by Cutuli, 2014). These two emotion-regulation strategies have also been shown to mediate the connection between music engagement and wellbeing outcomes with cognitive reappraisal predicting positive outcomes and expressive suppression predicting negative ones (Chin & Rickard, 2014).

Preliminary evidence shows that capacities for perceiving and expressing emotion in music relate to general emotional skills (e.g., Resnicow et al., 2004; Wöllner, 2012). Saarikallio et al. (2014) studied this in adolescents and showed that typical musical expressions (e.g., using slow tempo for sadness) were related to high scores in empathy, while incongruent musical expressions (e.g., staccato articulation for sadness) were related to high scores in behavioural problems. Meanwhile, Davis and colleagues (2020) showed that general emotional intelligence (as measured by Mayer-Salovey-Caruso Emotional Intelligence Test–Youth Version [MSCEIT-YV]; Mayer et al., 2005) did not correlate with recognition of vocal emotions in general, but high scores in MSCEIT-YV subscales for perceiving emotions and managing emotions were related to the tendency to more rarely choose anger as the representative emotion of the vocal expressions. Overall, however, knowledge concerning adolescents’ emotional expression through musical body movement, or its linkages to their broader emotional skills, is scarce, and the findings are scattered among a few studies conducted with diverse measures.

## Rationale, aims and hypotheses

Despite the wide acknowledgement of the embodied nature of emotions, there is little empirical research on how young people embody their emotional expressions in music. Prior literature on musical expression and music-related body movement has mainly been conducted with adults, some with children, little with adolescents. The current study was designed to investigate the features of music-related body movement that adolescents would use to express emotions.

The combination of music and movement provides a context in which adolescents’ embodied emotional expression can be studied in a relatively naturalistic setting. The musical instrument we selected for the current study was a djembe drum. We chose this instrument for several reasons. Djembe is a rope-tuned skin-covered goblet drum, used prominently by the Mandinka or Malinké people of West Africa. Played with bare hands, the djembe has been used in West Africa for centuries to tell emotional stories with rhythm. As such, a skilled drummer is considered one who “can make the djembe talk.” Djembe can produce a wide variety of sounds, making it a highly versatile drum. Yet, it is an easy instrument to approach, and since it is played with the hands, it requires no special skills to produce sound from. The fact that djembe is traditionally used to create emotional stories (as opposed to, say, as a signalling drum) made it ideally suited to this study, since emotional qualities were precisely what we sought to elicit from our participants.

The first aim of the study was to investigate how different features of adolescents’ embodied musical expressions, while playing the djembe drum, would relate to the expression of given emotions. Each of the four quadrants of the dimensional model of emotion was represented by the selected emotions: Other quadrants included one emotion each (happiness, tenderness, sadness), while the high-arousal low-valence quadrant included two emotions (anger and fear). Hypotheses were grounded on the dimensional model, but the inclusion of both fear and anger allowed additional exploration of potential within-quadrant differences. In particular, we expected the following:

- Speed: Since both general movement research and music and movement research have connected faster movement speed with high arousal (Amaya et al., 1996; Castellano et al., 2007; Paterson et al., 2000; Pollick et al., 2001; Punkanen et al., 2017; Wallbott, 1998), we hypothesised that movement speed/pace would be higher when expressing the high-arousal emotions of anger, fear and happiness than for expressing the low-arousal emotions of sadness and tenderness.
- Acceleration: Since music and movement research has connected greater variance/acceleration of movement with high arousal (Saarikallio et al., 2013), we hypothesised that movement variance/acceleration would be higher when expressing the high-arousal emotions of anger, fear and happiness than when expressing the low-arousal emotions of sadness and tenderness
- Length: Since prior music and movement and general movement research have demonstrated a connection between greater amount of movement and positive emotions (Dael et al., 2012; Luck et al., 2010) and since we can generally expect a preference for positive emotional experiences, we hypothesised that the performance time/duration would be longer when expressing positive emotions than when expressing negative emotions. Furthermore, due to the aforementioned hypothesis of high arousal relating to higher speed, we expected the performances to possibly take longer when expressing low-arousal versus high-arousal emotions. Hence, the greatest differences in performance length were expected to be observed between the expressions of tenderness and anger as well as between tenderness and fear, with tenderness expression being longer in duration than anger or fear expression.
- Area: Since prior research has demonstrated that positive emotions relate to open postures (Castellano et al., 2007; Coulson, 2004; De Silva & Bianchi-Berthouze, 2004; Schouwstra & Hoogstraten, 1995; Wallbott, 1998) and approach motivation relates to wider movement (Amaya et al., 1996; Paterson et al., 2000; Pollick et al., 2001; Wallbott, 1998), we hypothesised that the area/space/breadth of the movement would be larger when expressing positive emotions than when expressing negative emotions, particularly when expressing high-arousal positive emotions. Hence, the greatest difference was expected between happiness and sadness, with happiness being expressed via a larger area/space than sadness.

As a second aim, we explored whether the bodily expressions of basic emotions would relate to adolescents’ broader emotional skills and tendencies. We therefore correlated the movement expressions with self-report scores on cognitive reappraisal and expressive suppression (ERQ, Gross & John, 2003). Since there is very little research on connections between adolescents’ emotion embodiment and cognitive emotional competence, this part of the study remained exploratory. However, we hoped to establish some preliminary knowledge on how embodied emotion expression and cognitive-behavioural emotion regulation may be connected in this age group.

## Method

### Participants

Data were collected from a total of 60 adolescents (43 female, 17 male, mean age = 14.72 years, *SD* = 0.45). Around half of the adolescents had received some musical instrument or singing lessons for at least one year including several instruments (piano [*n* = 12], guitar [*n* = 4], violin [*n* = 4], drums [*n* = 3], flute [*n* = 3], accordion [*n* = 1], horn [*n* = 1], voice [*n* = 1], nondisclosed [*n* = 2]). Participants were recruited through local schools. The first author of the article visited the schools to contact eighth graders, inform them about the study and recruit volunteers. Participation was rewarded with a cinema voucher. All participants and their guardians gave their informed consent prior to their inclusion in the study and were free to discontinue the experiment at any point. The ethics board of the University of Jyväskylä gave the ethical permission for the study.

### Procedure and self-report measures

The current study was conducted as part of a larger day of experiments within a research project that investigated adolescents’ emotion-related responses to music. Measures for the current study consisted of a djembe task and a survey task, conducted in separate sessions. In the djembe task, the participants were recorded individually. They were asked to wear a black motion-capture suit to which reflective mocap markers were attached. After that, they familiarised themselves with the lab, wearing the markers, and were introduced to the djembe. They were shown and practised playing it together with one of the experimenters until they felt comfortable. During actual data collection, they performed by themselves without the experimenter and were asked to express the following emotions through drumming: happiness, anger, sadness, tenderness and fear. Participants could choose the order of these emotions by themselves and would indicate the respective emotion by saying it aloud before starting to drum. Participants were free to start and stop as they liked, no minimum or maximum duration was provided by the experimenters. There was no further contact with the experimenter, as the experimenter stepped out of the participant’s visual field behind a wall panel, while the participant performed the emotions. There was no feedback given, apart from a response that the emotion name was received and that the recording was running. For the survey task, participants were asked to complete a battery of questionnaires including demographic questions and the ERQ (Gross & John, 2003). The ERQ is a 10-item self-report instrument assessing cognitive reappraisal (e.g., *When I want to feel more positive emotion, I change the way I’m thinking about the situation*) and expressive suppression (e.g., *I control my emotions by not expressing them*). Items are answered on a 7-point Likert-type scale ranging from 1 (*strongly disagree*) to 7 (*strongly agree*). The Reappraisal scale consists of six items, and the Suppression scale consists of four items. Cronbach’s alphas for both scales in the current data were at acceptable levels; .84 for Reappraisal and .70 for Suppression.

### Apparatus for the movement measures

**Figure 1.**
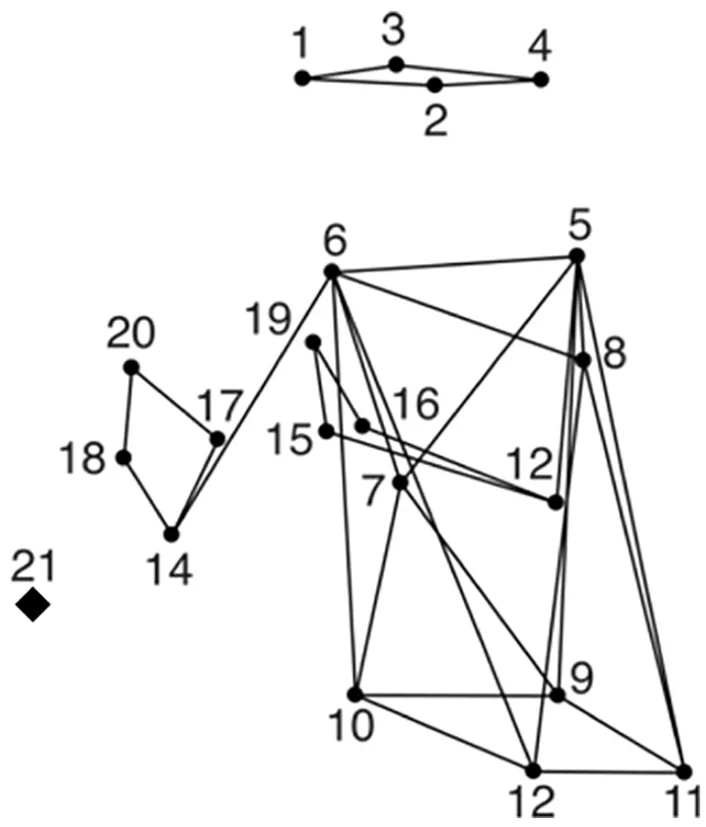
Marker Locations. *Note*. Djembe marker indicated with a diamond.

Participants’ movements while djembe drumming were recorded using an eight-camera optical infrared-based motion capture system (Qualisys ProReflex, Qualisys Track Manager software), tracking the three-dimensional positions of 21 reflective markers at a frame rate of 120 Hz. Twenty markers were attached to each participant’s upper body, while one marker was placed at the top end of the djembe for reference. The locations of the markers can be seen in Figure 1 and were as follows (L = left, R = right, F = front, B = back)—1: LF head; 2: RF head; 3: LB head; 4: RB head; 5: L shoulder; 6: R shoulder; 7: sternum; 8: spine (T5); 9: LF hip; 10: RF hip; 11: LB hip; 12: RB hip; 13: L elbow; 14: R elbow; 15: L wrist/radius; 16: L wrist/ulna; 17: R wrist/radius; 18: R wrist/ulna; 19: L middle finger; 20: R middle finger; 21 upper rim of djembe. The djembe was equipped with a contact microphone to directly, and with as little distortion as possible, record the drumming. Furthermore, the room sound was recorded with two overhead microphones positioned at a height of approximately 2.5 m. These microphones, the djembe microphone and the synchronisation pulse transmitted by the Qualisys cameras were recorded into ProTools software in order to synchronise the motion capture data with the drumming afterwards and for reference purposes. In addition, four Sony video cameras were used to record the sessions for reference purposes.

### Movement feature extraction

The recorded data were labelled using Qualisys Track Manager software and subsequently exported in .tsv format. In order to extract various kinematic features, the exported files were imported into MATLAB via the Motion Capture (MoCap) Toolbox (Burger & Toiviainen, 2013). Using MoCap Toolbox, the data were first divided into the five emotions and trimmed to start with the first hit and stop with the final hit. As we were mainly interested in hand movements during the drumming, we focused the analysis on wrist and finger markers. However, as those three markers were highly correlated due to the hand being a relatively rigid unit while hand drumming, selecting one marker seemed a parsimonious step to take. Since infrared-based motion capture is an optical method, we occasionally lost marker data if the marker was not visible to the cameras, resulting in missing frames and thus gaps in the data. In order to avoid gaps as much as possible, we checked the percentage of gaps of the two wrist markers and the finger marker of each hand in each performance and chose the marker (separately for each hand and performance) that had the fewest missing frames. Thus, the analysis was based on the finger, radius or ulna marker of each hand. At this stage, nine participants were excluded, due to more than 15% gaps in at least one of the five performances (5), technical errors while collecting the data (2) or missing questionnaire data (2). Therefore, the final analysis was conducted on 51 adolescents (37 female, 14 male, mean age = 14.74 years, *SD* = 0.47).

Thereafter, a set of 14 movement features relating to the hypotheses and previous studies was extracted from the hand position data to cover different movement characteristics, body parts and movement types. These features included the following: Mean Hand Speed, *SD* Hand Speed, Mean Hand Acceleration, *SD* Hand Acceleration, Mean Hand Jerk, *SD* Hand Jerk, Mean Drum Area, *SD* Drum Area, Fluidity, Performance Length, Hits, Hits/second, Dispersion of Hits, Dynamic Variation. A detailed description of each extracted feature is presented in Supplemental Appendix 1.

### Movement feature analysis

Given that several of these movement descriptors, in particular, the speed, acceleration and jerk variables, correlated to a high degree, Principal Component Analysis (PCA) was run on these 14 movement features to reduce the number of features and find a set of unique movement variables. Using the Kaiser criterion (eigenvalue of component larger than 1), four principal components were retained, accounting for 83.87% of the variance of the original data (PC 1: 39.87% variance explained after rotation; PC 2: 15.55%; PC 3: 15.10%; PC 4: 13.35%). Subsequently, the solution was rotated using Varimax rotation with Kaiser normalisation to distribute the variance across the four components more evenly and maximise orthogonality between components. The rotated component loadings are shown in Table 1. Instead of interpreting the loadings per component and continuing with the rotated scores in the further analysis, we opted to use the original variables that received the highest loading per component to remain closer to the actual movement descriptors and retain interpretability of the movements—PC 1: *SD* Hand Acceleration; PC 2: Hits/sec; PC 3: Performance Length; PC 4: Mean Drum Area. These four movement features were used in the subsequent analysis, in which repeated-measure analyses of variance (ANOVAs) were conducted to investigate how the movement features differed across the different emotion expressions. Furthermore, Spearman’s rank-order correlations were calculated between the movement features and the Reappraisal and Suppression scores.

**Table 1.** Component loadings of the rotated component matrix.

Feature	Component			
	1	2	3	4
Mean Hand Speed	.872	−.112	.159	.332
*SD* Hand Speed	.920	.089	.060	.291
Mean Hand Accel.	.922	−.302	.148	.112
***SD* Hand Accel.**	**.982**	−.060	.077	.079
Mean Hand Jerk	.904	−.369	.111	.045
*SD* Hand Jerk	.979	−.094	.044	.049
**Mean Drum Area**	.388	.021	−.024	**.840**
*SD* Drum Area	.191	−.044	.066	.792
Fluidity	−.350	.592	−.078	.510
**Performance Length**	.013	.276	**.935**	.046
Hits	.118	−.187	.926	−.014
**Hits/sec**	.168	**−.858**	.102	−.135
Dispersion of Hits	−.041	.793	−.010	−.190
Dynamic Variation	−.176	.298	−.538	−.017

Highest loading per component marked in bold.

## Results

### Differences in movement characteristics when expressing emotions

**Figure 2.**
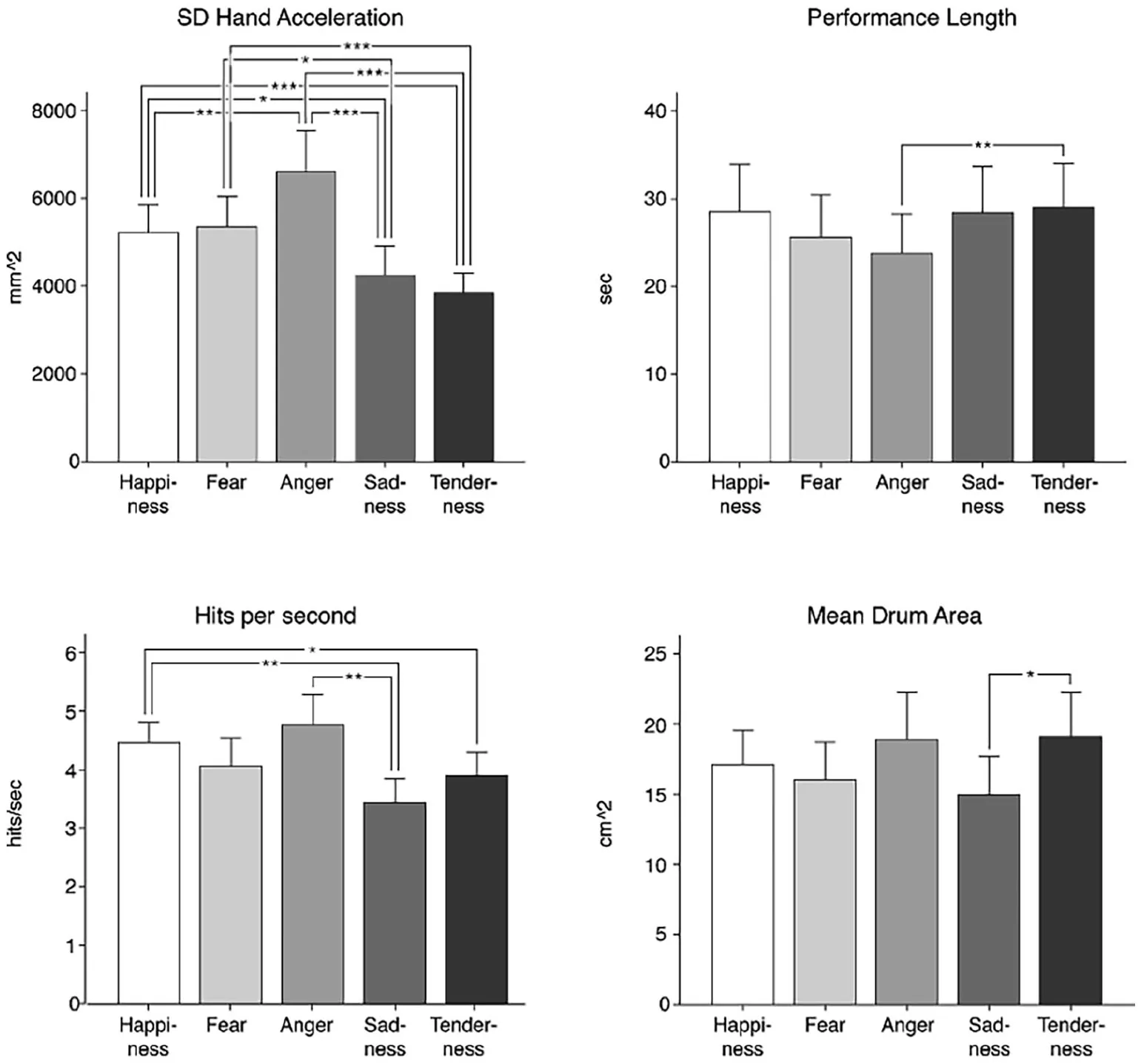
Movement feature averages per emotion with indication of significant differences in pairwise comparisons. *Note.* Error bars represent 95% CI. *p < .05, **p < .01, ***p < .001.

In order to investigate whether the adolescents used different movement characteristics in line with our hypotheses, repeated-measures ANOVAs were run on each of the four movement features. Greenhaus-Geisser corrected values are reported where the result of Mauchly’s test indicated sphericity of the data. All four analyses returned a significant main effect: *SD* Hand Acceleration: *F*(2.63, 131.24) = 15.17, *p* = .000, *η*^2^*_G_* = .23; Performance Length: *F*(4, 200) = 3.11, *p* = .013, *η*^2^*_G_* = .06; Hits/sec: *F*(3.06, 153.11) = 5.98, *p* = .001, *η*^2^*_G_* = . 11; Mean Drum Area: *F*(3.31, 165.38) = 2.71, *p* = .041, *η*^2^*_G_* = .05. A graphical illustration is provided in Figure 2.

To follow up these main effects, pairwise comparisons using Bonferroni correction were conducted. The mean values of hits/s were highest for anger (*M* = 4.76, *SD* = 1.84), followed by happiness (*M* = 4.46, *SD* = 1.25), fear (*M* = 4.06, *SD* = 1.73), tenderness (*M* = 3.90, *SD* = 1.45) and sadness (*M* = 3.43, *SD* = 1.48). Significant differences between the emotions were found between happiness and sadness (*p* = .002) as well as between happiness and tenderness (*p* = .035), with happiness having more hits per second than both sadness and tenderness and both anger and sadness (*p* = .009), with anger having more hits per second than sadness. These results support hypothesis 1 regarding movement speed being faster for high- compared to low-arousal emotions.

For *SD* Hand Acceleration, anger (*M* = 6,603.44, *SD* = 3,354.78) received the highest average value, that is, most varied acceleration while drumming, across adolescents, followed by fear (*M* = 5,351.18, *SD* = 2,478.72), happiness (*M* = 5,219.47, *SD* = 2,230.72), sadness (*M* = 4,239.14, *SD* = 2,426.44) and tenderness (*M* = 3,847.73, *SD* = 1,588.21). Significant differences were found between the performances for happiness and anger (*p* = .008), with anger being expressed with higher variation in hand acceleration than happiness; happiness and sadness (*p* = .04) as well as happiness and tenderness (*p* = .001), with happiness being expressed with higher variation in hand acceleration in both cases; fear and sadness (*p* = . 02) as well as fear and tenderness (*p* = .001), with fear being expressed with higher variation in hand acceleration in both cases; and finally anger and sadness (*p* = .001) as well as anger and tenderness (*p* = .001) with anger being expressed with higher variation in hand acceleration in both cases. The results support hypothesis 2 concerning movement variance/acceleration being greater for high-arousal than for low-arousal emotions. In addition, anger was expressed with higher levels of acceleration than happiness.

Regarding Performance Length, happiness (*M* = 28.50, *SD* = 19.16), sadness (*M* = 28.40, *SD* = 18.64) and tenderness (*M* = 28.95, *SD* = 17.95) received very similar average values across participants, while fear (*M* = 25.60, *SD* = 17.29) and especially anger (*M* = 23.75, *SD* = 16.15) were expressed in less time. However, only one significant difference was observed: between anger and tenderness (*p* = .004), with anger being performed significantly more quickly than tenderness. These results offer some support for hypothesis 3 concerning the duration of the performance being longer for positive valence and for low arousal, although the results were only significant for perhaps the most extreme pair of emotions studied (Tenderness – Anger).

For Mean Drum Area, adolescents covered the largest drum area when expressing tenderness (*M* = 19.10, *SD* = 11.26) and anger (*M* = 18.90, *SD* = 12.09), followed by happiness (*M* = 17.10, *SD* = 8.75), fear (*M* = 16.00, *SD* = 8.75) and sadness (*M* = 15.00, *SD* = 9.65). Only the difference between tenderness and sadness (*p* = .037) was found to be significant, with a larger area covered for tenderness than for sadness. For hypothesis 4, the results thus remain inconclusive. They do provide support for the hypothesis of drum space/area being larger for expressing positive than negative emotions – but only between tenderness and sadness. The hypothesis of drum area being larger for high-arousal emotions was not supported.

### Connection of emotion expression to emotion regulation

In order to investigate whether adolescents’ tendencies to regulate their emotions were reflected in their movement patterns while drumming, the four movement features were correlated (using Spearman’s rank-order correlation) with the results from the ERQ (Gross & John, 2003) separately for the four emotions (Table 2). While the Suppression scale did not reveal any significant correlations with the movement features, the Reappraisal scale did. It showed significant negative correlations with *SD* Hand Acceleration for expression of happiness, fear, sadness and tenderness. Thus, adolescents scoring high on Reappraisal used more stable acceleration patterns (i.e., were hitting the drum more evenly in relation to force). Hits/s showed a significant negative correlation with Reappraisal for the expression of fear and anger, implying that high Reappraisal adolescents hit fewer times per second when performing fear and anger than low Reappraisal adolescents. Performance Length showed significant negative correlations with Reappraisal for all emotion expressions – happiness, fear, anger, sadness and tenderness, suggesting that adolescents high on Reappraisal required less time to express any of the four emotions than adolescents low on Reappraisal. Related to Mean Drum Area, Reappraisal scores correlated significantly negatively with sadness and tenderness, with the negative correlations indicating that high-Reappraisal adolescents used a smaller drum area than adolescents low on Reappraisal when expressing sadness and tenderness. These correlations were performed without correcting for multiple comparisons, as the nature of this analysis was exploratory, and we were interested in a broad overview of correlation patterns. A full correlation table including the results for both Reappraisal and Suppression can be found in Table 2.

**Table 2.** Correlations between movement features and the Emotion Regulation Questionnaire scores.

	*SD* Hand Acceleration									
	Happiness		Fear		Anger		Sadness		Tenderness	
	*r*	*p*	*r*	*p*	*r*	*p*	*r*	*p*	*r*	*p*
**Reappraisal**	**−.288**	**.040**	**−.338**	**.015**	−.212	.135	**−.356**	**.010**	**−.359**	**.010**
**Suppression**	−.131	.359	−.044	.761	−.220	.121	−.127	.373	−.042	.770
	Performance Length									
	Happiness		Fear		Anger		Sadness		Tenderness	
	*r*	*P*	*r*	*p*	*r*	*p*	*r*	*p*	*r*	*p*
**Reappraisal**	**−.327**	**.019**	**−.280**	**.047**	**−.337**	**.015**	**−.367**	**.008**	**−.381**	**.006**
**Suppression**	−.132	354	.040	.778	−.122	.393	−.201	.158	−.105	.464
	Hits/sec									
	Happiness		Fear		Anger		Sadness		Tenderness	
	*r*	*p*	*r*	*p*	*r*	*p*	*r*	*p*	*r*	*p*
**Reappraisal**	−.101	.482	**−.386**	**.005**	**−.331**	**.018**	.024	.869	.235	.097
**Suppression**	−.183	.200	−.079	.583	−.262	.064	.012	.932	−.030	.832
	Mean Drum Area									
	Happiness		Fear		Anger		Sadness		Tenderness	
	*r*	*p*	*r*	*p*	*r*	*p*	*r*	*p*	*r*	*p*
**Reappraisal**	−.258	.067	−.049	.731	−.090	.529	**−.287**	**.041**	**−.297**	**.034**
**Suppression**	−.070	.625	−.180	.206	−.105	.465	−.144	.312	−.203	.154

Significant correlations are indicated in bold.

## Discussion

Our aims in the present study were two-fold: First, to investigate how different movement features exhibited in playing a djembe drum would relate to the expressions of basic emotions representing the four quadrants of the dimensional model, and second, to explore how these expressions would correlate with the adolescents’ general emotion-regulatory competence. The results of the study provide evidence that adolescents can elaborately embody emotional expressions through musical play. The emotion expressions were very much in line with our hypotheses concerning the dimensional emotion model: greater speed and acceleration of movement were related to higher arousal, while larger area and longer performance duration were related to more positive valence. In addition, however, some differences between fear and anger were observed even though the two emotions represent the same quadrant of the dimensional model.

*Speed* (as measured by hits per second) was particularly reflective of the arousal dimension: Happiness and anger differed significantly from sadness (and happiness also from tenderness), making this feature rather reflective of arousal than valence. Such connection of fast movement to the expression of high-arousal emotions has been evidenced by a broad range of prior research (Amaya et al., 1996; Burger et al., 2013; Castellano et al., 2007; Paterson et al., 2000; Pollick et al., 2001; Saarikallio et al., 2013; Wallbott, 1998). Fear did not significantly differ from anger, but unlike anger and happiness, fear did not differ from the low-arousal emotions. This suggests that while anger and fear represent the same quadrant, their expressions may still differ, and such difference may reflect, for instance, the approach‒avoidance dimension (the increase vs. decrease of behavioural activation; Elliot & Thrash, 2002), which potentially becomes manifested in higher speed for anger.

*Acceleration variance* revealed a slightly more complex pattern: It was particularly reflective of anger and fear. Anger did not significantly differ from fear, the second-highest scoring emotion, but it did score significantly higher than the other emotions. In line with our arousal-related hypothesis, the lowest-scoring emotions were the “soft” low-arousal emotions of sadness and tenderness, which did not differ from each other but scored significantly lower than anger, fear and happiness. We did not have a hypothesis about valence for this movement feature but still observed a difference between anger and happiness. The finding has resonance to how anger and fear as specific emotions have been characterised in previous research. Prior music research has shown that expression of anger contains rhythmic features such as sudden rhythmic changes (e.g., syncopations), accelerando and sharp contrasts between “long” and “short” notes and that fear contains large tempo variability, jerky rhythms, large timing variability and large micro-structural irregularity (Juslin & Laukka, 2004). It has also been shown that when people move to music that is rated to be expressive of anger, their body movement contains a lot of acceleration/jerk (Burger et al., 2013). It may be that acceleration variance is reflective of anger and fear as combinations of *both* high arousal and negative valence.

Regarding *performance length*, we had a dual hypothesis, expecting performance length to reflect both positive valence and low arousal. The results supported this, concerning only one emotion pair reflective of the *combination* of these dimensions: Tenderness (positive, low-arousal emotion) scored significantly higher than anger (negative, high-arousal emotion). It may be that in the case of sadness and happiness, one emotion dimension counteracts the impact of the other (for sadness, the negative valence counteracts low arousal; for happiness, the high arousal counteracts positive valence). This suggests that in future research, it is important to consider the potential combinatory/interaction effects of the different affective dimensions. Overall, performance length is not a commonly studied feature in music or movement research, and some caution is needed, since alternative explanations may exist. For instance, whether our adolescents particularly liked expressing tenderness or whether they found it difficult to express and therefore needed some time for it remains an open question.

Regarding the *area* of movement, we had a slightly different dual hypothesis: Greater use of the drum area was expected to relate to positive valence and high arousal. However, the only significant difference observed was between tenderness and sadness, with tenderness characterised by broader area use, which indicates that drum area is used to differentiate the emotional valence between low-arousal emotions. It is possible that our findings rather reflect the approach‒avoidance motivation difference than the valence‒arousal dimensions per se. Approach motivation is known to relate to broader movement (Amaya et al., 1996; Castellano et al., 2007; Paterson et al., 2000; Pollick et al., 2001; Wallbott, 1998) as well as more open postures (Castellano et al., 2007; Coulson, 2004; De Silva & Bianchi-Berthouze, 2004; Saarikallio et al., 2013; Schouwstra & Hoogstraten, 1995; Wallbott, 1998). The three highest-scoring emotions of happiness, anger and tenderness all represent approach motivation, while the two lowest-scoring emotions of sadness and fear represent avoidance motivation. However, this does not explain why sadness did not differ from the other approach motivation emotions of anger and happiness. One alternative explanation may be that the larger area used for tenderness results from the performance length: a longer performance simply grants more time to use different areas of the drum. Another alternative explanation is that the movement area is not a meaningful measure for assessing the emotional expression in djembe playing as there is relatively little room to vary the performance compared to, for instance, whole-body dance movement.

Our results reveal clear patterns in how certain movement features are used to express emotional dimensions and emotions that are reflective of the combinations of these dimensions. Indeed, if addressing the results from the viewpoint of specific emotions, we can conclude the following: *Happiness* is separated from the low-arousal emotions of tenderness and sadness by its faster speed and higher acceleration variation, but it shows no significant differences with fear and its only difference to anger is having lower acceleration variation than anger. *Anger* is characterised by having high acceleration variation, and it also has higher speed than sadness and shorter performance length than tenderness. *Fear* is perhaps the most difficult emotion to distinguish from the other emotions using these movement features; it only significantly differs from sadness and tenderness by higher acceleration variation. *Sadness* has a relatively distinctive expression pattern. It differs from anger, fear and happiness by lower acceleration variation and from anger and happiness by lower speed, and it differs from tenderness by smaller area used. Finally, *tenderness* also demonstrates significant differences with all other emotions. It has lower acceleration variation than anger, fear and happiness, it has lower speed than happiness, it is performed longer than anger and it is expressed through broader area than sadness. Overall, the findings lend support to the idea that core affect dimensions of valence and arousal are a good overall framework for understanding emotional expression in music (Cespedes-Guevara & Eerola, 2018) and that adolescents are highly skilful in using a variety of movement features for this. However, anger and fear, which represent the same quadrant of the model, also showed slightly different patterns in their relations to other emotions, which suggests that there is also room for further granularity and nuances to be investigated. Future research could include more systematic comparisons of more than one emotion selected from the same quadrant of the dimensional model of emotion (e.g., also happiness vs. pride, sadness vs. boredom), to test – challenge or confirm – the dimensional and constructionist propositions in further detail.

Our second aim was to explore how these embodied emotion expressions of adolescents might relate to their general emotion-regulatory tendencies. Correlations were only found for reappraisal, not for suppression. We wish to remain cautious about interpreting the correlations, and therefore only address the clearest general trends involving almost all emotions. Higher reappraisal was related to shorter performances and more stable acceleration patterns. Shorter and more stable performances may indicate that young people high on reappraisal are somehow more aware and confident of how to perform the different emotions. We could say that they knew better what they were doing. This is in line with previous research showing connections between emotion regulation and emotion differentiation skills (Barrett & Gross, 2001; Feldman Barrett et al., 2001) and between musical emotion expression and the broader social-emotional skill of cognitive empathy (Saarikallio et al., 2014). It seems plausible to argue that the use of reappraisal relates to greater capacity for differentiating between the emotions and therefore also to expressing them musically. This is also supported by recent intervention research that has generated promising results in advancing adolescents’ general emotion-regulation skills through a music-listening intervention that focused on discussing music’s emotional content and recognising emotions in music (Tuned In; Dingle & Fay, 2017). The current study adds one additional level of depth to this discussion by focusing on body movement. The embodied level of affective knowledge and the conceptual/cognitive level of affective knowledge are perhaps connected to each other. Our findings encourage future research, particularly on how the different levels of affective knowledge interact and contribute to each other, and whether causal links can be evidenced. Can we, for instance, gain better conceptual insight of emotions and then further their management or use in social interaction by training non-verbal emotion expressions through music and art?

Certain limitations of the study need to be addressed. While the djembe is an expressive drum, it only focuses on rhythmical musical expression. Other musical features, such as timbre and harmony, are therefore not included in the scope of the current design. Musical expression containing these other elements would naturally allow an even more elaborate expression of different emotional nuances. The study is also similarly restricted concerning movement expression: the design focused on features expressed by hands while sitting and playing – a restricted setting compared to, say, full-body movement in dance. One concern is also that even if fear were not included in the analyses, the inclusion of it in the expressions performed may have somehow biased the expression of other emotions. Furthermore, while *N* = 60 is a high number for a motion capture laboratory experiment, it is a small sample for studying individual differences. It is also important to note that the mocap measures are performative, while ERQ measures traits. To connect these levels is somewhat risky because the sensitivity of mocap is very high, while self-reports on a few items are a coarse instrument. The correlations with ERQ are therefore discussed only at the level of the clearest trends. Finally, in terms of emotion-regulation tendencies, the current study focused only on cognitive reappraisal and expressive suppression, while future work might also be interested in other strategies such as acceptance, avoidance and rumination, which are also known to relate to youth mental health (Schäfer et al., 2016). Emotion regulation also is only one aspect of youth emotional development, and other aspects such as empathy or body awareness could also be studied in future work, if a design feasibly allows a larger sample size.

In conclusion, young people seem to have an elaborate capacity to embody emotional qualities in their musical expression, and their expressions are very much in line with those exhibited by adults. Our results highlight adolescents as being competent in expressing emotions at the embodied level, and our study corroborates motion capture as a method to objectively measure and quantify this complex non-verbal level of emotional knowledge. Our findings are a pioneering step that encourages further investigation into how the embodied aspects of emotional insight may relate to other aspects of emotional development. They may also provide grounds for a dialogue with practitioners who work to support adolescents’ emotional growth through creative, art-based methods.

## Supplemental Material

sj-docx-1-pom-10.1177_03057356241257426 – Supplemental material for Embodiment of emotions in adolescents’ musical expression Supplemental material, sj-docx-1-pom-10.1177_03057356241257426 for Embodiment of emotions in adolescents’ musical expression by Suvi Saarikallio, Birgitta Burger and Geoffrey Luck in Psychology of Music

## References

[bibr1-03057356241257426] Amaya K. Bruderlin A. Calvert T. (1996). Emotion from motion. In Proceedings of the graphics interface conference (pp. 228–229). Canadian Human-Computer Communications Society. 10.20380/GI1996.26

[bibr2-03057356241257426] Arom S. (1991). African polyphony and polyrhythm: Musical structure and methodology. Cambridge University Press. 10.1017/CBO9780511518317

[bibr3-03057356241257426] Ball G. Breese J. (2000). Emotion and personality in a conversational agent. In Cassel J. Sullivan J. Prevost S. Churchill E. (Eds.), Embodied conversational agents (pp. 189–219). MIT Press.

[bibr4-03057356241257426] Barrett L. F. Gross J. (2001). Knowing what you’re feeling and knowing what to do about its: Mapping the relation between emotion differentiation and emotion regulation. Cognition and Emotion, 15(6), 713–724. 10.1080/02699930143000239

[bibr5-03057356241257426] Berking M. Wupperman P. (2012). Emotion regulation and mental health: Recent findings, current challenges, and future directions. Current Opinion in Psychiatry, 25(2), 128–134. 22262030 10.1097/YCO.0b013e3283503669

[bibr6-03057356241257426] Boone R. T. Cunningham J. (2001). Children’s expression of emotional meaning in music through expressive body movement. Journal of Nonverbal Behavior, 25, 21–41. 10.1023/A:1006733123708

[bibr7-03057356241257426] Bowling D. L. Sundararajan J. Han S. Purves D. (2012). Expression of emotion in Eastern and Western music mirrors vocalization. PLOS ONE, 7(3), Article e31942. 10.1371/journal.pone.0031942 PMC3303771 22431970

[bibr8-03057356241257426] Burger B. Saarikallio S. Luck G. Thompson M. R. Toiviainen P. (2013). Relationships between perceived emotions in music and music-induced movement. Music Perception, 30(5), 517–533. 10.1525/mp.2013.30.5.517

[bibr9-03057356241257426] Burger B. Toiviainen P. (2013). MoCap Toolbox – A Matlab toolbox for computational analysis of movement data. In Bresin R. (Ed.), Proceedings of the 10th sound and music computing conference (pp. 172–178). KTH Royal Institute of Technology.

[bibr10-03057356241257426] Burger B. Toiviainen P. (2020). See how it feels to move: Relationships between movement characteristics and perception of emotions in dance. Human Technology, 16(3), 233–256. 10.17011/ht/urn.202011256764

[bibr11-03057356241257426] Camurri A. Mazzarino B. Ricchetti M. Timmers R. Volpe G. (2004). Multimodal analysis of expressive gesture in music and dance performances. In Camurri A. Volpe G. (Eds.), Lecture notes in computer science: Vol. 2915. Gesture-based communication in human-computer interaction (pp. 20–39). Springer. 10.1007/978-3-540-24598-8_3

[bibr12-03057356241257426] Carr E. W. Kever A. Winkielman P. (2018). Embodiment of emotion and its situated nature. In Newen A. De Bruin L. Gallagher S. (Eds.), The Oxford handbook of 4E cognition (pp. 529–552). Oxford University Press. 10.1093/oxfordhb/9780198735410.013.30

[bibr13-03057356241257426] Castellano G. Mortillaro M. Camurri A. Volpe G. Scherer K. (2008). Automated analysis of body movement in emotionally expressive piano performances. Music Perception, 26(2), 103–120. 10.1525/mp.2008.26.2.103

[bibr14-03057356241257426] Castellano G. Villalba S. D. Camurri A. (2007). Recognising human emotions from body movement and gesture dynamics. In Paiva A. C. R. Prada R. Picard R. W. (Eds.), Lecture notes in computer science: Vol. 4738. Affective computing and intelligent interaction (pp. 71–82). Springer. 10.1007/978-3-540-74889-2_7

[bibr15-03057356241257426] Cespedes-Guevara J. Eerola T. (2018). Music communicates affects, not basic emotions – A constructionist account of attribution of emotional meanings to music. Frontiers in Psychology, 9(215), 1–19. 10.3389/fpsyg.2018.00215 29541041 PMC5836201

[bibr16-03057356241257426] Chin T. Rickard N. S. (2014). Emotion regulation strategy mediates both positive and negative relationships between music uses and well-being. Psychology of Music, 42(5), 692–713. 10.1177/0305735613489916

[bibr17-03057356241257426] Coulson M. (2004). Attributing emotion to static body postures: Recognition accuracy, confusions, and viewpoint dependence. Journal of Nonverbal Behavior, 28, 117–139. 10.1023/B:JONB.0000023655.25550.be

[bibr18-03057356241257426] Crane E. A. Gross M. (2007). Motion capture and emotion: Affect detection in whole body movement. In Paiva A. C. R. Prada R. Picard R. W. (Eds.), Lecture notes in computer science: Vol. 4738. Affective computing and intelligent interaction (pp. 95–101). Springer. 10.1007/978-3-540-74889-2_9

[bibr19-03057356241257426] Cutuli D. (2014). Cognitive reappraisal and expressive suppression strategies role in the emotion regulation: An overview on their modulatory effects and neural correlates. Frontiers in Systems Neuroscience, 8, 175. 10.3389/fnsys.2014.00175 25285072 PMC4168764

[bibr20-03057356241257426] Dael N. Mortillaro M. Scherer K. R. (2012). Emotion expression in body action and posture. Emotion, 12(5), 1085–1101. 10.1037/a0025737 22059517

[bibr21-03057356241257426] Dahl S. Friberg A. (2007). Visual perception of expressiveness in musicians’ body movements. Music Perception, 24(5), 433–454. 10.1525/mp.2007.24.5.433

[bibr22-03057356241257426] Darwin C. (1872). The expression of the emotions in man and animals. John Murray.

[bibr23-03057356241257426] Davis S. K. Morningstar M. Dirks M. A. Qualter P. (2020). Ability emotional intelligence: What about recognition of emotion in voices? Personality and Individual Differences, 160, 109938.

[bibr24-03057356241257426] Denham S. A. Bassett H. Mincic M. Kalb S. Way E. Wyatt T. Segal Y. (2012). Social–emotional learning profiles of preschoolers’ early school success: A person-centered approach. Learning and Individual Differences, 22, 178–189. 22408363 10.1016/j.lindif.2011.05.001 PMC3294380

[bibr25-03057356241257426] De Silva P. R. Bianchi-Berthouze N . (2004). Modeling human affective postures: An information theoretic characterization of posture features. Computer Animation and Virtual Worlds, 15, 269–276. 10.1002/cav.29

[bibr26-03057356241257426] Dingle G. A. Fay C. (2017). Tuned In: The effectiveness for young adults of a group emotion regulation program using music listening. Psychology of Music, 45(4), 513–529. 10.1177/0305735616668586

[bibr27-03057356241257426] Edey R. Yon D. Dumontheil I. Press C. (2020). Association between action kinematics and emotion perception across adolescence. Journal of Experimental Psychology: Human Perception and Performance, 46(7), 657–666. 32584128 10.1037/xhp0000737

[bibr28-03057356241257426] Eerola T. Friberg A. Bresin R. (2013). Emotional expression in music: Contribution, linearity, and additivity of primary musical cues. Frontiers in Psychology, 4, Article 487. 10.3389/fpsyg.2013.00487 PMC3726864 23908642

[bibr29-03057356241257426] Eerola T. Vuoskoski J. (2013). A review of music and emotion studies: Approaches, emotion models, and stimuli. Music Perception, 30(3), 307–340. 10.1525/MP.2012.30.3.307

[bibr30-03057356241257426] Elliot A. J. Thrash T. M. (2002). Approach–avoidance motivation in personality: Approach and avoidance temperaments and goals. Journal of Personality and Social Psychology, 82(5), 804–818. 10.1037//0022-3514.82.5.804 12003479

[bibr31-03057356241257426] Feldman Barrett L. Gross J. Christensen T. C. Benvenuto M . (2001). Knowing what you’re feeling and knowing what to do about it: Mapping the relation between emotion differentiation and emotion regulation. Cognition and Emotion, 15(6), 713–724. 10.1080/02699930143000239

[bibr32-03057356241257426] Gabrielsson A. Lindström E. (2010). The role of structure in the musical expression of emotions. In Juslin P. N. Sloboda J. A. (Eds.), Handbook of music and emotion: Theory, research, applications (pp. 367–400). Oxford University Press.

[bibr33-03057356241257426] Georgiou E. Mai S. Pollatos E. (2016). Describe your feelings: Body illusion related to alexithymia in adolescence. Frontiers in Psychology, 7, Article 1690. 10.3389/fpsyg.2016.01690 PMC5083713 27840618

[bibr34-03057356241257426] Gray J. A. (1970). The psychophysiological basis of introversion–extroversion. Behaviour Research and Therapy, 8(3), 249–266. 10.1016/0005-7967(70)90069-0 5470377

[bibr35-03057356241257426] Gross J. J. (2002). Emotion regulation: Affective, cognitive, and social consequences. Psychophysiology, 39(3), 281–291. 12212647 10.1017/s0048577201393198

[bibr36-03057356241257426] Gross J. J. John O. P. (2003). Individual differences in two emotion regulation processes: Implications for affect, relationships, and well-being. Journal of Personality and Social Psychology, 85(2), 348–362. 10.1037/0022-3514.85.2.348 12916575

[bibr37-03057356241257426] Gross J. J. Levenson R. W. (1993). Emotional suppression: Physiology, self-report and expressive behavior. Journal of Personality and Social Psychology, 64(6), 970–986. 10.1037//0022-3514.64.6.970 8326473

[bibr38-03057356241257426] Halberstadt A. G. Denham S. A. Dunsmore J. C. (2001). Affective social competence. Social Development, 10, 79–119.

[bibr39-03057356241257426] Herbert R. Dibben N. (2018). Making sense of music: Meanings 10- to 18-year-olds attach to experimenter-selected musical materials. Psychology of Music, 46(3), 375–391. 10.1177/0305735617713118

[bibr40-03057356241257426] Hietanen J. K. Glerean E. Hari R. Nummenmaa L. (2016). Bodily maps of emotions across child development. Developmental Science, 19(6), 1111–1118. 10.1111/desc.12389 26898716

[bibr41-03057356241257426] Juslin P. N. (2013). What does music express? Basic emotions and beyond. Frontiers in Psychology, 4, Article 596. 10.3389/fpsyg.2013.00596 PMC3764399 24046758

[bibr42-03057356241257426] Juslin P. N. Laukka P. (2004). Expression, perception, and induction of musical emotions: A review and a questionnaire study of everyday listening. Journal of New Music Research, 33(3), 217–238. 10.1080/0929821042000317813

[bibr43-03057356241257426] Kluft E. S. Poteat J. Kluft R. P. (1986). Movement observations in multiple personality disorder: A preliminary report. American Journal of Dance Therapy, 9, 31–46. 10.1007/BF02274237

[bibr44-03057356241257426] Koppensteiner M. Grammer K. (2010). Motion patterns in political speech and their influence on personality ratings. Journal of Research in Personality, 44(3), 374–379. 10.1016/j.jrp.2010.04.002

[bibr45-03057356241257426] Lazarus R. S. Alfert E. (1964). Short-circuiting of threat by experimentally altering cognitive appraisal. Journal of Abnormal Psychology, 69(2), 195–205. 10.1037/h0044635 14213291

[bibr46-03057356241257426] Leman M. Maes PJ. Nijs L. Van Dyck E. (2018). What is embodied music cognition?. In Bader R. (Ed.), Springer handbook of systematic musicology. Springer Handbooks. Springer, Berlin, Heidelberg. 10.1007/978-3-662-55004-5_34

[bibr47-03057356241257426] Luck G. Saarikallio S. Burger B. Thompson M. R. Toiviainen P. (2010). Effects of the Big Five and musical genre on music-induced movement. Journal of Research in Personality, 44(6), 714–720. 10.1016/j.jrp.2010.10.001

[bibr48-03057356241257426] Mayer J. D. Salovey P. (1997). What is emotional intelligence? In Salovey P. Sluyter D. J. (Eds.), Emotional development and emotional intelligence: Educational implications (pp. 3–34). Basic Books.

[bibr49-03057356241257426] Mayer J. D. Salovey P. Caruso D. R. (2005). The Mayer-Salovey-Caruso Emotional Intelligence Test – Youth version (MSCEIT-YV) [Research version]. Multi Health Systems.

[bibr50-03057356241257426] Nawrot E. S. (2003). The perception of emotional expression in music: Evidence from infants, children and adults. Psychology of Music, 31(1), 75–92. 10.1177/0305735603031001325

[bibr51-03057356241257426] Nettl B. (2000). An ethnomusicologist contemplates universals in musical sound and musical culture. In Wallin N. L. Merker B. Brown S. (Eds.), The origins of music (pp. 463–472). MIT Press. 10.7551/mitpress/5190.003.0032

[bibr52-03057356241257426] Newen A. Gallagher S. de Bruin L. (2018). 4E cognition: Historical roots, key concepts, and central issues. In Newen A. de Bruin L. Gallagher S. (Eds.), The Oxford handbook of 4E cognition (pp. 3–18). Oxford University Press. 10.1093/oxfordhb/9780198735410.013.1

[bibr53-03057356241257426] North A. C. Hargreaves D. J. O’Neill S. A. (2000). The importance of music to adolescents. British Journal of Educational Psychology, 70(2), 255–272. 10.1348/000709900158083 10900782

[bibr54-03057356241257426] Paterson H. M. Pollick F. E. Sanford A. J. (2000). The role of velocity in affect discrimination. In Moore J. D. Stenning K. (Eds.), Proceedings of the twenty-third annual conference of the cognitive science society (pp. 756–761). Psychology Press.

[bibr55-03057356241257426] Pollick F. E. Paterson H. M. Bruderlin A. Sanford A. J. (2001). Perceiving affect from arm movement. Cognition, 82(2), B51–B61. 10.1016/S0010-0277(01)00147-0 11716834

[bibr56-03057356241257426] Punkanen M. Saarikallio S. Leinonen O. Forsblom A. Kulju K. Luck G. (2017). Emotions in motion: Depression in dance-movement and dance-movement in treatment of depression. In Karkou V. Oliver S. Lycouris S. (Eds.), The Oxford handbook of dance and wellbeing (pp. 839–862). Oxford University Press. 10.1093/oxfordhb/9780199949298.013.58

[bibr57-03057356241257426] Resnicow J. E. Salovey P. Repp B. H. (2004). Is recognition of emotion in music performance an aspect of emotional intelligence? Music Perception, 22(1), 145–158. 10.1525/mp.2004.22.1.145

[bibr58-03057356241257426] Rickard N. (2004). Intense emotional responses to music: A test of the physiological arousal hypothesis. Psychology of Music, 32(4), 371–388. 10.1177/0305735604046096

[bibr59-03057356241257426] Russell J. A. (1980). A circumplex model of affect. Journal of Personality and Social Psychology, 39(6), 1161–1178. 10.1037/h0077714

[bibr60-03057356241257426] Saarikallio S. Erkkilä J. (2007). The role of music in adolescents’ mood regulation. Psychology of Music, 35(1), 88–109. 10.1177/0305735607068889

[bibr61-03057356241257426] Saarikallio S. Luck G. Burger B. Thompson M. Toiviainen P. (2013). Dance moves reflect current affective state illustrative of approach-avoidance motivation. Psychology of Aesthetics, Creativity, and the Arts, 7(3), 296–305. 10.1037/a0032589

[bibr62-03057356241257426] Saarikallio S. Vuoskoski J. Luck G. (2014). Adolescents’ expression and perception of emotion in music reflects their broader abilities of emotional communication. Psychology of Well-Being, 4(1), 1–16. 10.1186/s13612-014-0021-8

[bibr63-03057356241257426] Saarni C. (1999). The development of emotional competence. Guilford Press.

[bibr64-03057356241257426] Salimpoor V. N. Benovoy M. Longo G. Cooperstock J. R. Zatorre R. J. (2009). The rewarding aspects of music listening are related to degree of emotional arousal. PLOS ONE, 4(10), Article e7487. 10.1371/journal.pone.0007487 PMC2759002 19834599

[bibr65-03057356241257426] Schäfer J. Naumann E. Holmes E. Tuschen-Caffier B. Samson A. (2016). Emotion regulation strategies in depressive and anxiety symptoms in youth: A meta-analytic review. Journal of Youth and Adolescence, 46(2), 261–276. 10.1007/s10964-016-0585-0 27734198

[bibr66-03057356241257426] Schouwstra S. J. Hoogstraten J. (1995). Head position and spinal position as determinants of perceived emotional state. Perceptual and Motor Skills, 81(2), 673–674. 10.2466/pms.1995.81.2.673 8570377

[bibr67-03057356241257426] Ter Bogt T. F. M. Vieno A. Doornwaard S. M. Pastore M. van den Eijnden R. J. J. M . (2017). “You’re not alone”: Music as a source of consolation among adolescents and young adults. Psychology of Music, 45(2), 155–171. 10.1177/0305735616650029

[bibr68-03057356241257426] Thompson M. R. Luck G. (2011). Exploring relationships between pianists’ body movements, their expressive intentions, and structural elements of the music. Musicae Scientiae, 16(1), 19–40. https://doi.org/10.1177%2F1029864911423457

[bibr69-03057356241257426] Troje N. F. (2008). Retrieving information from human movement patterns. In Shipley T. F. Zacks J. M. (Eds.), Understanding events: From perception to action (pp. 308–334). Oxford University Press.

[bibr70-03057356241257426] Van Beveren M. L. Goossens L. Volkaert B. Grassmann C. Wante L. Vandeweghe L. Verbeken S. Braet C . (2019). How do I feel right now? Emotional awareness, emotion regulation, and depressive symptoms in youth. European Child and Adolescent Psychiatry, 28(3), 389–398. 10.1007/s00787-018-1203-3 30069654

[bibr71-03057356241257426] Van Zijl A. G. W. Luck G . (2013). Moved through music: The effect of experienced emotions on performers’ movement characteristics. Psychology of Music, 41(2), 175–195. 10.1177/0305735612458334

[bibr72-03057356241257426] Wallbott N. (1998). Bodily expression of emotion. European Journal of Social Psychology, 28(6), 879–896. 10.1002/(SICI)1099-0992(1998110)28:6<879::AID-EJSP901>3.0.CO;2-W

[bibr73-03057356241257426] Ward D. Stapleton M. (2012). Es are good: Cognition as enacted, embodied, embedded, affective and extended. In Paglieri F. (Ed.), Consciousness in interaction: The role of the natural and social context in shaping consciousness (pp. 89–104). John Benjamins.

[bibr74-03057356241257426] Wöllner C. (2012). Is empathy related to the perception of emotional expression in music? A multimodal time-series analysis. Psychology of Aesthetics, Creativity, and the Arts, 6(3), 214–223. 10.1037/a0027392

[bibr75-03057356241257426] Zeman J. Cassano M. Perry-Parrish C. Stegall S. (2006). Emotion regulation in children and adolescents. Journal of Developmental & Behavioral Pediatrics, 27(2), 155–168. 16682883 10.1097/00004703-200604000-00014

