# Supplementary material for: Embodiment of emotions in adolescents’ musical expression
Source: Psychol Music. 2024 Oct 8;54(5):661–79. doi: 10.1177/03057356241257426 (PMC13568481; doi:10.1177/03057356241257426)
Supplement: Supplementary file 1 [file sj-docx-1-pom-10.1177_03057356241257426.docx]

**Appendix 1.** **Descriptions of the originally extracted 14 movement features**

- Mean Hand Speed: arithmetic mean of the norm of the instantaneous velocity (i.e., the first time derivative of position) of the averaged hand markers per participant and performance.
- SD Hand Speed: standard deviation of the norm of the instantaneous velocity of the averaged hand markers per participant and performance.
- Mean Hand Acceleration: arithmetic mean of the norm of the instantaneous acceleration (i.e., the second time derivative of position) of the averaged hand markers per participant and performance.
- SD Hand Acceleration: standard deviation of the instantaneous acceleration of the norm of the averaged hand markers per participant and performance.
- Mean Hand Jerk: arithmetic mean of the norm of the instantaneous jerk (i.e., the third time derivative of position) of the averaged hand markers per participant and performance.
- SD Hand Jerk: standard deviation of the instantaneous jerk of the norm of the averaged hand markers per participant and performance.
- Mean Drum Area: arithmetic mean of the projection of the trajectory of the hand markers on the horizontal surface of the djembe drum, averaged across four-second analysis windows with a two-second overlap, i.e., how much of the drum surface they covered while playing.
- SD Drum Area: standard deviation of the projection of the trajectory of the hand markers on the horizontal surface of the djembe drum, averaged across four-second analysis windows with a two-second overlap.
- Fluidity: overall movement fluidity/smoothness measure based on the ratio of velocity to acceleration. The combination of high velocity and low acceleration reflects smooth movement, whereas the combination of low velocity and high acceleration reflects non-smooth movement.
- Performance Length: total length in seconds of each performance.
- Hits: total number of hits during the performance, based on acceleration peaks.
- Hits/second: number of hits per second, based on acceleration peaks.
- Dispersion of Hits: standard deviation of temporal distances between hits, based on acceleration peaks. The higher the standard deviation, the more varied/irregular rhythms were drummed.
- Dynamic Variation: standard deviation of the heights of the acceleration peaks. The higher the peak, the more strength/force was used to hit the djembe resulting in a louder sound; thus, the higher the standard deviation, the more variation in strength (i.e., dynamics) of hits occurred.
